# Mechanical consequences at the tendon-bone interface of different medial row knotless configurations and lateral row tension in a simulated rotator cuff repair

**DOI:** 10.1186/s40634-022-00536-1

**Published:** 2022-09-19

**Authors:** Carlos Maia Dias, Sérgio B. Gonçalves, António Completo, Manuel Ribeiro da Silva, Clara de Campos Azevedo, Jorge Mineiro, Frederico Ferreira, João Folgado

**Affiliations:** 1 Department of Bioengineering, and iBB - Institute for Bioengineering and Biosciences, Instituto Superior Técnico, Universidade de Lisboa, Lisbon, Portugal; 2 Hospital CUF Tejo, Shoulder and Elbow Unit, Lisbon, Portugal; 3 IDMEC, Instituto Superior Técnico, Universidade de Lisboa, Lisbon, Portugal; 4 TEMA, Department of Mechanical Engineering, University of Aveiro (UA), Aveiro, Portugal; 5 Hospital CUF Porto, Porto, Portugal; 6 Life and Health Sciences Research Institute (ICVS), School of Medicine, University of Minho, Campus de Gualtar, Braga, Portugal; 7 ICVS/3B’s - PT Government Associate Laboratory, Braga/Guimarães, Portugal; 8 Hospital CUF Tejo, Elbow and Shoulder Unit, Lisbon, PT Portugal; 9 Hospital Dos SAMS de Lisboa, Lisbon, Portugal; 10 Hospital CUF Descobertas, Lisbon, Portugal

**Keywords:** Rotator cuff, Medial row, Contact force, Pressure, Area, Lateral row tension

## Abstract

**Purpose:**

Little is known about the direct influence of different technical options at the rotator cuff tendon-bone interface (TBI) and, more specifically, at the medial bearing row (MBR), regarding local contact force, area and pressure. We evaluated the mechanical repercussions of different medial row anchor configurations for that setting using different values of tension in the lateral row anchors.

**Methods:**

Knotless transosseous equivalent (TOE) rotator cuff repairs with locked versus nonlocked medial anchors and single versus double-hole suture passage were tested in a synthetic rotator cuff mechanical model, using 2 different values of lateral row tension. Contact force, area, pressure, peak force and MBR force were compared at the simulated TBI using a pressure mapping sensor.

**Results:**

When compared to locked anchors, medial row sliding configurations generate lower values for all the above-mentioned parameters.

The use of double-hole suture passage in the medial cuff generated slightly higher values contact area regardless of lateral row tension. At higher lateral row tension values, lower values of the remaining parameters, including MBR force, were found when compared to single-hole suture passage.

Lateral row anchor tension increase induced an increase of all parameters regardless of the medial row configuration and TBI contact force and MBR force were the most susceptible parameters, regardless of the medial row pattern.

**Conclusion:**

Medial row mechanism, suture configuration and lateral row tension interfere with the mechanical force, area and pressure at by TBI. Lateral row tension increase is a major influencer in those parameters.

These results can help surgeons choose the right technique considering its mechanical effect at the TBI.

**Supplementary Information:**

The online version contains supplementary material available at 10.1186/s40634-022-00536-1.

## Introduction

Tendon-bone healing failure / retear in rotator cuff repairs ranges from 0% [18] to 94% [14]. This complication is not only patient- and injury—related [26, 32], but also related to the surgical technique [18]. In medium and large sized tears, stiffer constructs such as transosseous-equivalent (TOE) repairs, are considered superior to less stiff ones [9, 10, 18, 34, 38, 44, 47] regarding integrity, clinical and biomechanical outcomes.

Despite those improved results, TOE repairs have also been associated to an increased risk of type 2 retears [5, 11, 21, 50], medial to the previous repair site, and harder to revise as the remaining tendon to reattach is shorter and more retracted than in type 1 retears. Stress overload in the medial bearing row (MBR) (an imaginary line that connects the most anterior and posterior sutures passed in the medial cuff), repair overtensioning, excessive suture medialization and increased tendon abrasion by suture material were some of the hypothetical causes for this phenomenon and for that reason, optimization of TOE surgical technique was recently recommended to reduce the risk of tendon hypoperfusion and decrease stress concentration in medial bearing row (MBR) [5, 45].

While overmedialization [24], abrasion of the suture material [12, 15, 23, 53] and repair overtensioning [2, 41] have all been investigated, knowledge on stress overload in the MBR is lacking, including from a biomechanical point of view.

To date, only one work [29] has evaluated the mechanical effect of different medial suture passage configurations in that tendon region, and none described the biomechanical implications at the tendon-bone interface (TBI) and at the MBR of using locked medial row anchors (anchors that do not allow sutures to slide in its eyelet) or sliding medial row anchors (anchors that allow its sutures to slide in its eyelet) in knotless TOE repairs.

This study thus aimed to compare the force applied in the MBR and the entire TBI contact force, contact pressure, contact area and peak force if two different medial row anchor mechanisms, two types of suture passages and two different lateral row tension values are used.

We hypothesized that all evaluated parameters value would increase if locked medial anchors were used and if lateral row tension values increased, while individual suture limb passage in the medial cuff (double-hole suture passage) would increase TBI contact area without increasing contact force or pressure, while decreasing the MBR contact force.

## Methods

### Experimental setup

#### Measured parameters and used materials

This was an experimental biomechanical study that evaluated total contact force, pressure and area, peak pressure and total force at the MBR using a Tekscan® 5051 pressure mapping sensor (Tekscan Inc.®, Boston, MA) in three different type of knotless TOE repairs [39]. The sensor has a flexible array of 46 × 46 force sensors with a spatial resolution of 62 sensors per cm^2^. In order to fit the area under the tendon model the sensor was folded and, following the manufacturers´ recommendation, we did not perforate it nor with needles nor sutures. The sensor maximum pressure was defined to 0.69 MPA, as we considered this to be a substantial high value of pressure that can surely induce tendon ischemia considering that this value is 39 times higher than the normal systolic blood pressure [3]. Its calibration was performed using a Shimadzu® calibrator (Shimadzu Corporation©, Kyoto, Japan).

To obtain homogeneous testing samples simulating tendon-bone interface we chose to use SAWBONES® SKU 1521–12–2 training model (SAWBONES®, Vashon, WA) instead of cadaveric tissue. This type of model consists of a rigid foam that mimics the mechanical properties of the humeral head and also includes a neoprene foam that replaces the tendon, albeit not trying to replicate its mechanical characteristics. SAWBONES models have been previously used by the medical and biomechanics community to perform their training and research activities, being considered a valid tool for comparative analysis when the biological aspects are not relevant or when they induce experimental variability (e.g. analysis of orientation of the acetabular cup in osteotomy techniques, anchor fixation testing and rotator cuff repair evaluation) [13, 17, 42].

#### Test groups

**Fig. 1:**
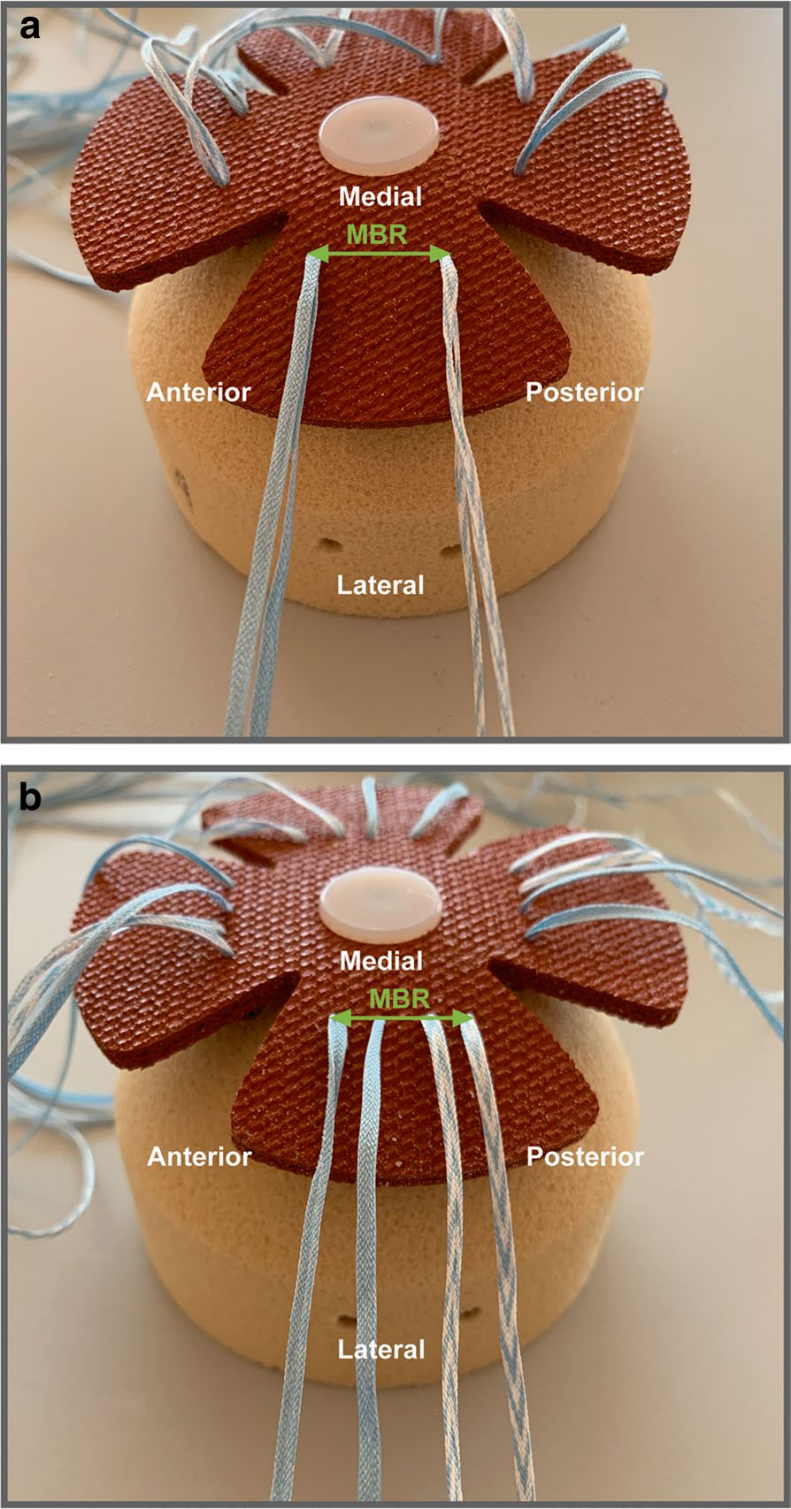
Representation of the medial row configuration and MBR (green): **a** Single hole suture passage (SP Group); **b** Double hole suture passage (DP and SLDP Groups)

Three different types of knotless TOE repairs were explored in this experiment (groups), each with a different combination of type medial passage configuration and medial row anchor mechanism. These groups were:

- DP group– Double-hole passage and locked anchor (DP);
- SLDP Group – Double-hole passage and sliding anchor (SLDP);
- SP Group – Single-hole passage and locked anchor (SP).

(see figs. 1a and b).

A fourth group (single passage with sliding anchors) was not added because that type of construct is rarely used in the clinical setting and for the purpose of evaluating the effect of medial row passage and type of medial row mechanism, the previous groups sufficed.

With these 3 groups we were able to evaluate and compare the mechanical effects at the TBI of using two different types of medial row mechanisms (one that allows suture tapes to slide in the anchors (SLDP) versus another that does not allow anchors to slide(DP)) and suture passage (both tape limbs from each medial row anchor pass in a single tendon hole (SP) in opposition to passing each tape limb individually in the tendon model (DP)).

To study medial row mechanism, we compared groups 1 (locked anchors—DP) and 2 (sliding anchors – SLDP) and to compare the types of medial passage we compared groups 1 (DP) and 3 (SP).

We did not compare all groups among themselves because our aim was not to rank the repairs, but to compare the effect at the tendon bone interface of specific surgical options between each other. We also did not compare groups 2 and 3 among themselves as they differed in both variables that we were studying.

Within each group, we also evaluated the effect of lateral row tension increase, in the case by increasing lateral row tension from 25 to 50 N.

#### Mock surgical technique description

For the medial row we used two locked 5.5 mm Footprint Ultra Pk anchors® (Smith & Nephew, London, UK), single-loaded with Ultratape® in groups 1 and 3. In its turn, in group 2 (SLDP), two Haelicoil® 5.5 mm anchors (Smith & Nephew, London, UK) also single-loaded with Ultratape® were chosen for that purpose. For the lateral row, two 5.5 mm Footprint Ultra PK® anchors (Smith & Nephew, London, UK) were used in all groups. A total of four anchors was used for each isolated trial.

Five trials using new sawbones model, new anchors and new suture limbs for each trial, were performed for each test group (*N* = 5).

As previously reported [29], a flexible plastic template was used to ensure that each anchor was placed in a consistent fashion and that all sutures had the same distance among them in each trial. We used the same single-sized needle for tape passage in each trial, regardless of the medial row configuration. The sensor was placed under the mock tendon and held with finger pressure. Both most anterior tape limbs of each medial anchor were pulled and placed in the anterolateral (AL) anchor with the sutures slacked to avoid undetermined tensioning. Sutures limbs were then individually tensioned using two suture tensioners (EU000715 Suture Tensioner, Smith and Nephew, London, UK®) previously calibrated using a Shimadzu® calibrator (Shimadzu Corporation©, Kyoto, Japan), which allow for the measurement of four different tension values: 25, 50, 75 and 100 N.

In each of the 3 groups, the sutures in the AL anchor were tensioned until the 25 N mark was reached. The anchor was then locked, and the tensioners released. The posterolateral (PL) anchor was then placed following the same sequential steps but using the most posterior suture limbs of each medial row anchor. Sensor finger stabilization was released when sufficient contact to the mechanical model allowed stable sensor position. After reaching the 25 N tension mark in the PL anchor suture limbs, the force map was acquired using the I-Scan Lite software (Tekscan Inc.®, Boston, MA).

**Fig. 2:**
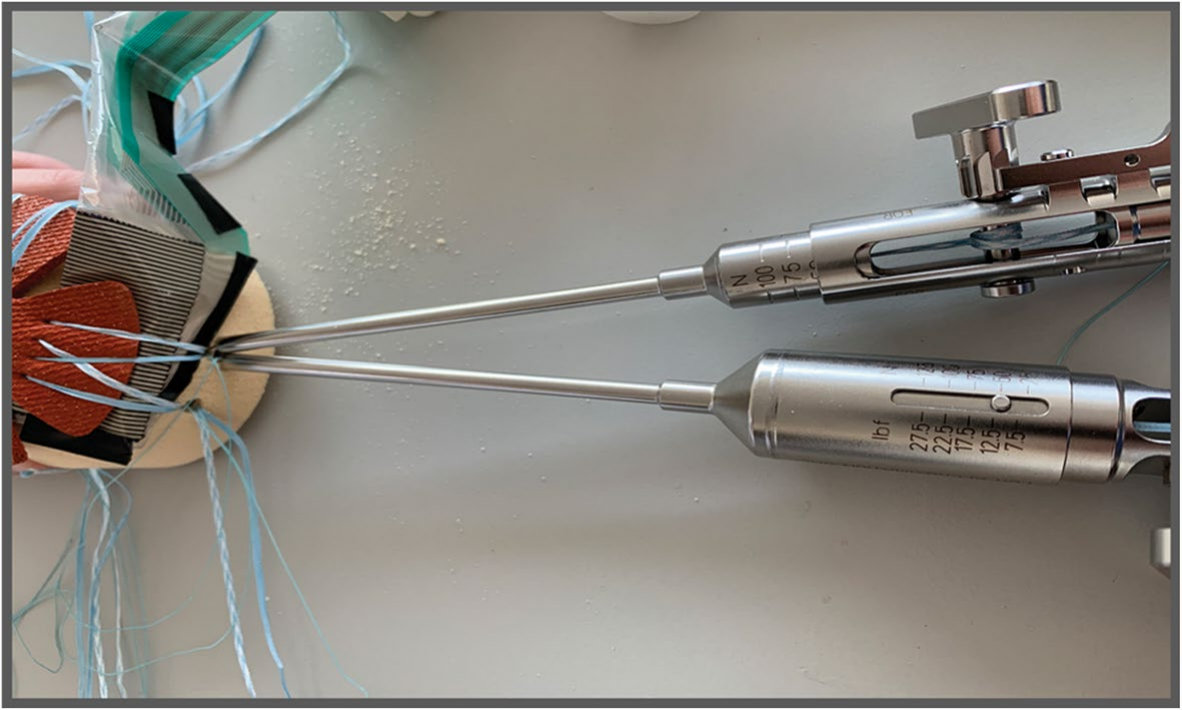
Representation of the experimental setup used for measuring the contact force, area and pressure in the model and the tension in the tapes for the SLDP configuration (please note the 50 N mark in the calibrated tensioners)

The lateral anchors were then unlocked, and all four suture limbs were slacked for reuse using the exact same mentioned methods, but this time, performing the lateral anchor locking at 50 N of lateral tension (Fig. 2).

We chose to evaluate the results at 25 and 50 N taking into consideration Park´s 90 N threshold [37], that if surpassed did not translate into further contact area to the tendon bone interface. Also, in a previous work [29] the use of 75 N of lateral tension generated TBI pressure values that largely exceeded the arterial and capillary pressure and in addition, we also experienced some anchor pullout at 75 N during our preliminary trials, so 25 and 50 N of lateral tension seemed adequate values for the purpose of this part of the study which was to assess the mechanical effect at the TBI of a specific and quantified increase in lateral row tension.

To increase trial homogeneity, all assemblies and tests were performed by the same shoulder fellowship trained surgeon with over 10 years of surgical experience.

### Data analysis

**Fig. 3:**
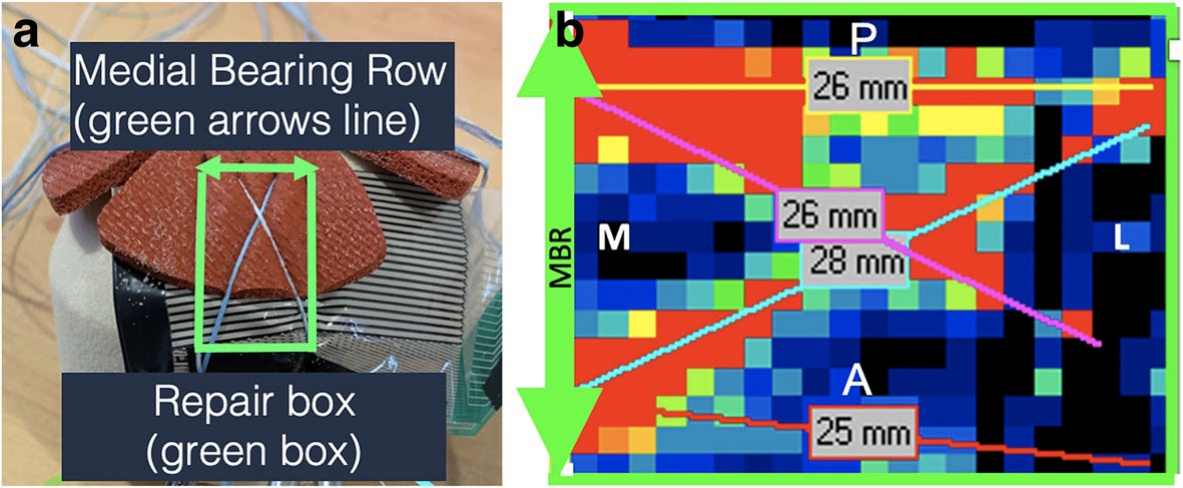
**a** Representation of the repair box (rectangle in green) and the Medial Bearing Row (arrowed green line); **b** Force mapping of the repair box (green rectangle), Medial Bearing Row (arrowed green line) and suture path (yellow, purple, light blue and red lines)

A repair box of 586 mm^2^ (27 × 21,85 mm), i.e., the region of analysis that simulate the TBI, was equally defined for each trial. The analysis of the contact force, area and pressure distribution, as well as maximum peak force for an area of sixteen (4 × 4) force cells (25.81mm^2^) were performed using I-Scan Lite® software. The same software was used to analyze the contact force applied in the MBR (defined as the most medial line of the previously described repair box (see figs. 1a, b, 3a and b).

The single cell saturation was set for 0.69 MPa, the maximum pressure applied during the calibration procedure.

### Statistical analysis

The type of medial row mechanism, medial suture configuration and lateral row tension values were considered independent variables in this work. Contact force, area, and pressure as well as peak force and MBR force were the dependent ones.

A post hoc power analysis using G*Power software v 3.1.9.7® was performed (see table S-1 and S-2).

A Mann–Whitney test with a null hypothesis that group results were similar was used to compare the 2 groups for medial row mechanism (comparison between DP and SLDP) and the 2 groups for medial row passage (DP vs SP).

The influence of the lateral tension increase within each group was analyzed using the Wilcoxon signed rank test.

The statistical analysis was performed on IBM SPSS Statistics v26 software (IBM, Armonk, NY). Statistical significance was set at *p* < 0.05, but we highlighted tendencies for three intervals: *p* ≤ 0.01 (*), 0.01 < *p* ≤ 0.05 (**) and 0.05 < *p* ≤ 0.1 (***).

## Results

Medial Row Mechanism: Locking (DP) vs. Sliding medial row anchors (SLDP).

**Fig. 4:**
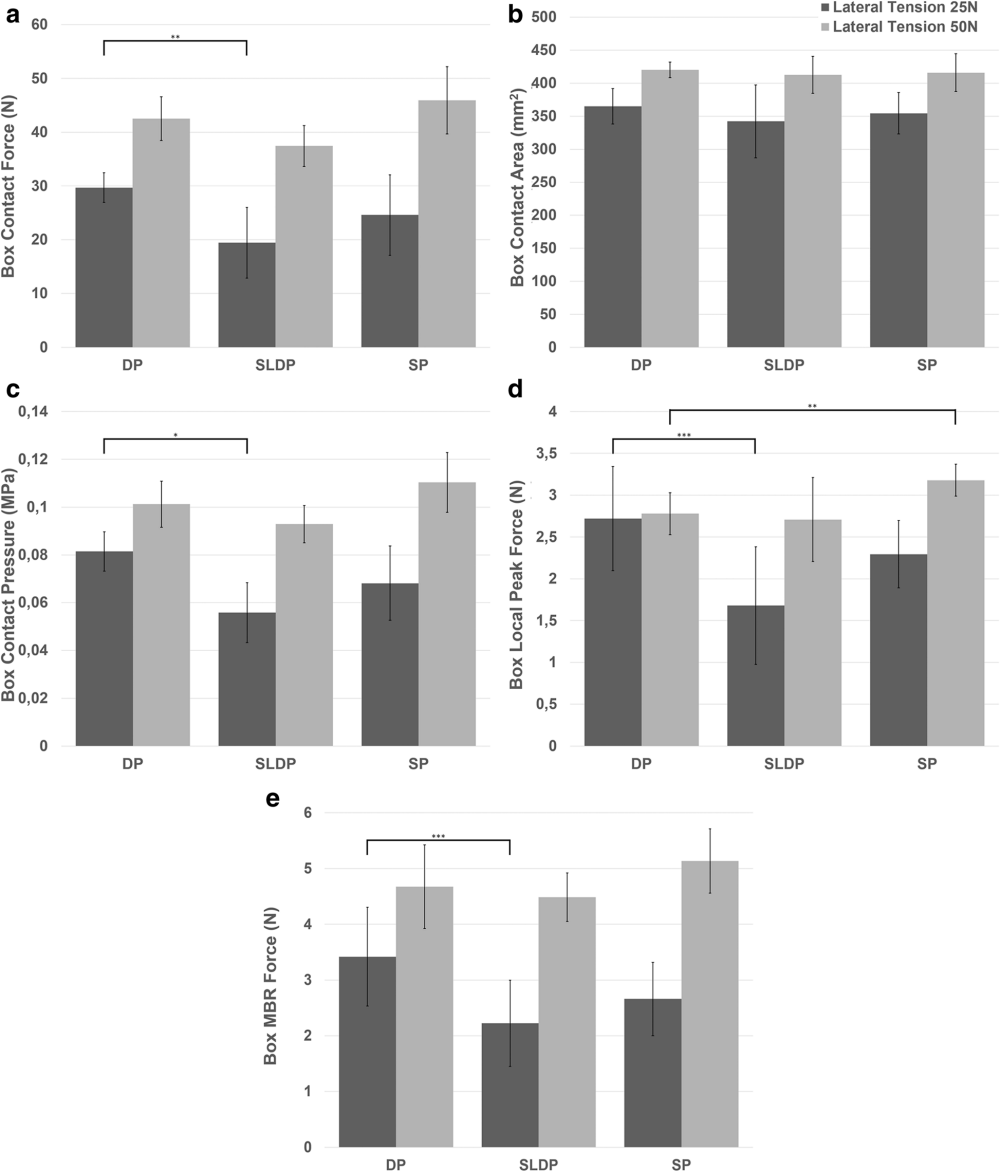
Comparison of the biomechanical outcomes for the repair box between the DP and SLDP groups and between the DP and SP groups for a value of lateral tension in the tapes of 25 N (dark gray) and 50 N (light gray): **a** Total contact force; **b** Contact area; **c** Contact Pressure; **d** Local Peak Force; **e** Total force in the MBR (*p* ≤ 0.01 (*), 0.01 < *p* ≤ 0.05 (**), 0.05 < *p* ≤ 0.1 (***))

Figure 4a-e summarizes results for all groups regarding the total contact force, pressure and contact area in the repair box according to the lateral row tension imposed in the assembly.

The use of locked anchors (DP) generated a higher mean contact force, area and pressure, irrespective of the applied lateral tension. However, significant differences were only obtained for the box force (*p* = 0.032) and pressure (*p* = 0.008) when a lateral tension of 25 N was used.

Local peak pressure and MBR force were also higher for the locked anchors for both tested tensions, with more notorious differences at lower lateral tension values (*p* < 0.10).

### Single-hole passage (SP) vs. double-hole passage (DP)

Regardless of the lateral tension applied, DP originated non-significant higher values of contact area.

At 25 N, DP achieved non-significant higher values for all studied parameters. However, when a lateral tension of 50 N was used, the SP group achieved higher values of contact force, pressure and MBR force, reaching statistical significance (*p* = 0.032) in local peak force (see Fig. 4a-e).

### Consequences of the increase in lateral row tension

**Fig. 5:**
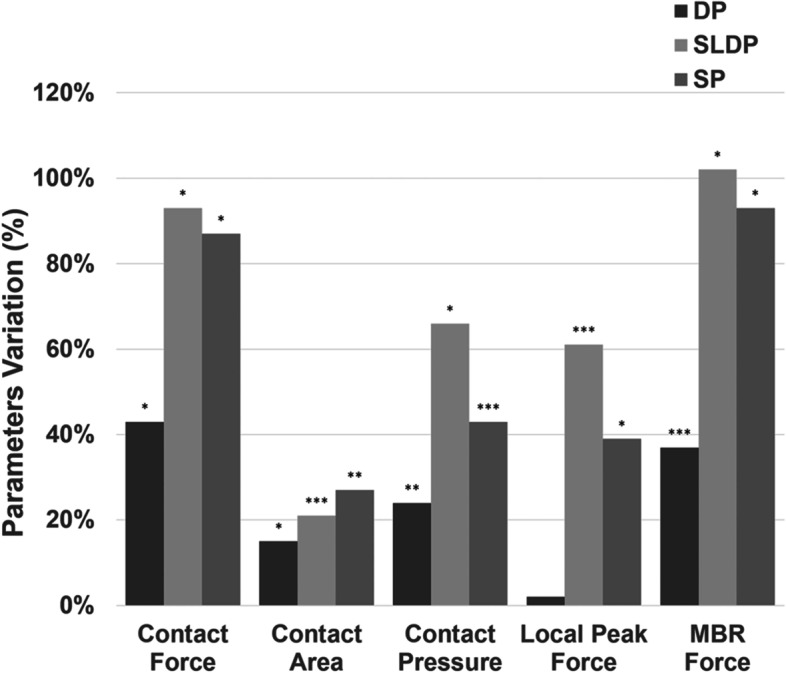
Variation of the biomechanical outcomes within each group for an increase of 100% (25 to 50 N) in lateral row tension (*p* ≤ 0.01 (*), 0.01 < *p* ≤ 0.05 (**), 0.05 < *p* ≤ 0.1 (***))

An increase in 100% of the lateral row tension resulted in significant variations in the contact force for all groups (Fig. 5), as well as contact area in the medial locked anchor groups (SP and DP) and pressure in the double-hole passage groups (DP and SLDP).

Nevertheless, in those groups in which the difference did not reach statistical significance, a tendency to increase was demonstrated.

MBR force also increased in all groups, but only significantly in SP and SLDP (*p* = 0.008).

Results also demonstrated that an increase in lateral row tension has a more pronounced effect in contact force and MBR force than in all other parameters, regardless of the group.

The obtained values for the biomechanical outcomes and statistical tests can be consulted in Supplementary Materials (Table S3-S7).

## Discussion

The main findings of our work are that the type of medial anchor mechanism and medial passage have a direct influence on the contact force, area and pressure as well as on peak force and MBR force at the TBI in knotless rotator cuff repairs. Moreover, careful attention should be provided to the amount of lateral row tension applied during surgery as this proved to have major impact in the mechanical parameters evaluated, including MBR force, regardless of the type of construct. The above-mentioned variables are the product of surgeon’s technical choices and technique and, in theory, can affect the rate and type of retear that can occur [11, 35, 50].

Most biomechanical studies to date aimed to evaluate the mechanical characteristics of the materials and rotator cuff assemblies, and their capacity to withstand deformation and failure at time 0 [1, 4, 8, 16, 20, 30, 33]. However, only a small number of reports have analyzed the mechanical consequences in terms of contact force, area and pressure at the tendon bone interface using pressure mapping sensors [30, 40, 46, 51, 52] while even less used controlled lateral tension for that purpose, despite its enormous relevance for the compressive effect of sutures at the TBI if transosseous equivalent are used [25, 37, 45].

Also, to the best of our knowledge, none compared medial sliding anchors to medial locked anchors, which justifies the relevance of this specific investigation.

When comparing the medial anchor mechanism (sliding vs. locked), the outcomes demonstrated that medial anchors with locked tapes tendentially generate higher mean contact force, area, and pressure, as well as peak forces and MBR force irrespective of the lateral tension applied.

These results are explained by the interaction between different forces in knotless rotator cuff tear repairs. According to Newton´s second law, a resultant force is the single force acting on the object when all the other individual forces have been combined. Literature has detailed how friction force generates an efficiency loss in a pulley, in such a way that in order to move an object on one side of the pulley, the tension force on the other side needs to be higher than the force acting on the object itself [36].

In the SLDP group, the medial sliding row acts like a (rigid) pulley and induces a loss of efficiency in the translation of lateral row pull force into compressive force at the TBI, probably explaining its lower values of contact force applied when comparing to the DP group, in which no medial pulley system exist. In this setting, the medial pulley friction force is removed from the net force equation, meaning all tension force and its vector of pull at the lateral row are counteracted only by that lateral anchor sliding mechanism and by the locked medial mechanism, generating a higher compressive force vector at the TBI both at lower and higher lateral tension values in this group when compared to SLDP.

It is important to note that the force transmitted in the SLDP pulley mechanism (${T}_{1}$) depends on the coefficient of friction $\left(\mu\right)$ and on the angle of contact in radians $\left(\beta\right)$ between the tape and the medial mechanism, and it can be calculated using the Capstan equation (also referred as Euler-Eytelwein equation) [49]:

$${T}_{2}={T}_{1}{e}^{\mu \beta }$$

in which ${T}_{2}$ is the tension applied by pulling the tapes [36, 48]. The purpose of this study was not to measure this specific medial mechanism friction force, nor could we do it with the available data, but our outcomes demonstrate its effect by showing the difference between DP and SLDP, and the previous explanation suffices.

Data regarding MBR contact force is also relevant as the SLDP group generated a non-significant lower force in that region when compared to DP, which can also probably be explained by the friction force effect on the medial sliding mechanism previously discussed. This can be clinically relevant because the MBR is the most stressed area of the repair [29] due to a high localized fixation strength [45], and stress reduction in this area can eventually help reduce the rates of type 2 retears [5, 45].

Regarding suture passage configuration, if larger lateral row tension values are used, a tendency for DP to confirm our initial hypothesis occurs, in which double-hole passage configurations increase the contact area without increasing the maximum force applied in the TBI when comparing to SP. This issue is of relevance as it can generate a lower contact pressure, which can have advantageous implications on the biological process of healing [45]. These results are aligned with our previous report [29], which demonstrated that in knotless TOE repairs with medial row sliding anchors, passing sutures individually (DP) significantly increases the contact area when compared to combined passage of suture limbs in a single pilot hole (SP).

Curiously, when compared to DP, the SP group generated higher MBR force at higher lateral tension values. This means that if higher lateral row tension is used, more force is applied per suture passage site at the MBR implying that contact force in those locations is clearly higher in the SP that in the DP group, generating uneven stress distribution that probably jeopardizes this important tendon area [5] and hypothetically increases the risk for type 2 retears [45].

Also as previously reported [29], results also demonstrated that at higher lateral tension values, single-hole passage in the medial cuff significantly increases peak force, which may provide higher focal stability but also hamper biological healing in that specific location [22], usually quite close or at the MBR [29]. Of specific interest, looking at McCarron et al. [31] description of failure in continuity in which regardless of tendon healing, some tissue retraction always occurs, excessive contact force at the MBR or near it prevents this phenomenon and can, hypothetically, increase the risk of type 2 retears.

Like Park et al.[37], Kummer [25] and Andre et al.[2], we also demonstrated that lateral row tension is one of the most important variables to be considered when performing any type of biomechanical evaluation at the TBI because it clearly impacts contact force, area and pressure, as well as MBR force, in all studied groups. Despite having significant differences between 25 and 50 N lateral tension, DP group was the most compliant one meaning that the increase in lateral tension translated into an increase of all studied variables but in a less pronounced manner than both SP and SLDP. In fact, the latter demonstrated the highest susceptibility to lateral row tension increase in all parameters except for area, in which SP superseded.

Our study has some strengths that should be highlighted. First, by avoiding the use of biological specimens, we obtained a more reproducible evaluation of the mechanical data, and reduced experimental variability, like reported by other authors [13, 17, 29, 43]. Second, the use of a template and a single sized needle for suture passage increased the homogeneity of anchor placement, suture passage location, and mock tendon damage. Third, by using an undamaged high-resolution sensor we were able to evaluate contact force, area and pressure with a more accurate method if compared to other published reports [9, 30, 40, 43, 46]. Fourth, we used a constant repair box, avoiding measurement of force in “no contact” regions of the sensor, which could approximate the pressure measurements by lowering the mean contact force. Fifth, to the best of our knowledge, this is the first report that evaluates the mechanical consequences at the TBI of using locked or sliding mechanisms in the medial row anchors and one of the very few specifically addressing the force applied in the MBR, and lastly, lateral row tensioning was measured and performed individually, which is the only way to accurately control lateral tension as using only one tensiometer to control tension in multiple sutures, if they have different initial tensions, which they usually do, the measured lateral tension corresponds only to the tauter suture limb.

The current study also presents some limitations. First, despite the post hoc statistical power analysis demonstrated that our sample was adequate for the evaluation of the effect of lateral row tension and for part of the dependent variable evaluation in the medial mechanism comparison (see supplemental tables 1 and 2), a small sample size is one of the drawbacks of this paper, as of most biomechanical reports [6, 7, 19, 27, 43]. The cost per trial, mainly driven by implant cost, was the major limiting factor for the sample number in this study and makes statistical power unobtainable for some comparisons that require over 400 trials.

Second, even though a single surgeon placed all the anchors and utilized a template so that their location would be reproducibly replicated, the angle and depth of placement of the medial anchors was not controlled. Considering that a constant lateral tension was applied, by changing both the angle at which the anchor enters the bone and its depth, the compressive force at the TBI, especially in the MBR, can change because the resultant compressive force depends on the angle between the pull force and the vertical axis of the anchor.

Also, as mentioned, friction force also interferes with the final compressive force and if the anchor is placed deeper, the tape can have a higher contact area with the bone and lower the resultant force.

Lastly, our mechanical model does not mimic tendon mechanical properties, but these are also quite variable between individuals and can even vary within the person according to its age, medical condition, medication anatomical location and use [28]. In fact, despite removing the biological variables, our model also precludes the immediate clinical setting translation of our results for that same reason.

## Conclusion

Knotless rotator cuff repairs generate a TBI contact force, area and pressure that is highly dependent on the lateral tension applied in the lateral anchors. Peak force and MBR force also increase if lateral row tension increases, especially in constructs that use medial sliding mechanism anchors. Despite that, when compared to locked configurations, these tend to generate lower values of all studied parameters. The adoption of single- or double-hole suture passages in the medial row also has mechanical consequences at the TBI as double-hole passage configurations consistently generate nonsignificant higher contact area, while reducing, force, pressure, peak force and MBR force if higher lateral row tension values are applied.

Despite the ideal biomechanical setting at the TBI is still to be established, our work can help surgeons decide which is the most adequate technique, when facing different patients or types of tears, although it is, unfortunately, insufficient to provide a critical analysis of the clinical consequences of these choices and to define the ideal compressive force at the medial bearing row to prevent type 2 retears.

## Supplementary Information

**Additional file 1:** **Table S1.** Statistical power analysis performed using G power software for comparisons of the dependent variables between groups. Considered acceptable statistical power if power > 0,75; Alpha error probability = 0,05.

**Additional file 2:** **Supplementary table S2.** Statistical power analysis performed using G power software for comparisons of tension variation within groups.

**Additional file 3:** **Supplementary Table S3.** Mean Comparison between locked medial anchor (DP) and sliding medial anchors (SLDP) regarding contact force, area and pressure, peak force and MBR force in the repair box (25N lateral tension) - * reached statistical significance. **Supplementary Table S4.** Mean comparison between locked medial anchor (DP) and sliding medial anchors (SLDP) regarding contact force, area and pressure, peak force and MBR force in the repair box (50N lateral tension) - * reached statistical significance. **Supplementary Table S5.** Mean comparison between tape double-hole passage (DP) and single-hole passage (SP) regarding contact force, area and pressure, peak force and MBR force in the repair box (25N lateral tension) - * reached statistical significance. **Supplementary Table S6.** Mean comparison between tape double–hole passage (DP) and single-hole passage (SP) regarding contact force, area and pressure, peak force and MBR force in the repair box (50N lateral tension) - * reached statistical significance. **Supplementary Table S7.** Variation within each group if lateral row tension increases 100% - * reached statistical significance.

## Data Availability

See supplementary material. All remaining data will be provided upon request to the corresponding author.

## Abbreviations

AL: Anterolateral
MBR: Medial bearing Row
PL: Posterolateral
TBI: Tendon Bone Interface
TOE: Transosseous equivalent
