# Supplementary material for: Mechanical consequences at the tendon-bone interface of different medial row knotless configurations and lateral row tension in a simulated rotator cuff repair
Source: J Exp Orthop. 2022 Sep 19;9:94. doi: 10.1186/s40634-022-00536-1 (PMC9482894; doi:10.1186/s40634-022-00536-1)
Supplement: Supplementary file 1 — Additional file 1: Table S1. Statistical power analysis performed using G power software for comparisons of the dependent variables between groups. Considered acceptable statistical power if power > 0,75; Alpha error probability = 0,05. [file 40634_2022_536_MOESM1_ESM.docx]

|  | | **Force** | | | **Area** | | | **Pressure** | | | **PFORCE** | | | **MBR** | | |
| --- | --- | --- | --- | --- | --- | --- | --- | --- | --- | --- | --- | --- | --- | --- | --- | --- |
|  |  | Power | Effect size | Ideal N | Power | Effect size | Ideal N | Power | Effect size | Ideal N | Power | Effect size | Ideal N | Power | Effect size | Ideal N |
| **SLDP - DP** | 25N | 0,78 | 2,02 | NA | 0,11 | 0,52 | 59 | 0,9 | 2,42 | NA | 0,56 | 1,56 | 13 | 0,49 | 1,43 | 15 |
| **DP - SP** |  | 0,22 | 0,89 | 35 | 0,08 | 0,36 | 211 | 0,3 | 1,07 | 25 | 0,75 | 1,98 | 43 | 0,25 | 0,96 | 31 |
|  |  |  |  |  |  |  |  |  |  |  |  |  |  |  |  |  |
| **SLDP - DP** | 50N | 0,25 | 1,29 | 31 | 0,07 | 0,34 | 231 | 0,25 | 0,95 | 32 | 0,05 | 0,17 | 872 | 0,07 | 0,31 | 289 |
| **DP - SP** |  | 0,14 | 0,64 | 67 | 0,05 | 0,19 | 231 | 0,19 | 0,81 | 46 | 0,67 | 1,89 | 10 | 0,15 | 0,68 | 118 |

Table S1- Statistical power analysis performed using G power software for comparisons of the dependent variables between groups. Considered acceptable statistical power if power > 0,75; Alpha error probability = 0,05
