# Supplementary material for: Mechanical consequences at the tendon-bone interface of different medial row knotless configurations and lateral row tension in a simulated rotator cuff repair
Source: J Exp Orthop. 2022 Sep 19;9:94. doi: 10.1186/s40634-022-00536-1 (PMC9482894; doi:10.1186/s40634-022-00536-1)
Supplement: Supplementary file 2 — Additional file 2: Supplementary table S2. Statistical power analysis performed using G power software for comparisons of tension variation within groups. [file 40634_2022_536_MOESM2_ESM.docx]

|  | **Force** | | | **Area** | | | **Pressure** | | | **PFORCE** | | | **MBR** | | |
| --- | --- | --- | --- | --- | --- | --- | --- | --- | --- | --- | --- | --- | --- | --- | --- |
|  | **Power** | **Effect size** | **Ideal N** | **Power** | **Effect size** | **Ideal N** | **Power** | **Effect size** | **Ideal N** | **Power** | **Effect size** | **Ideal N** | **Power** | **Effect size** | **Ideal N** |
| DP | 0,23 | 0,91 | 34 | 0,95 | 2,6 | 6 | 0,83 | 2,19 | 7 | 0,05 | 0,12 | 1861 | 0,54 | 1,53 | 13 |
| SLDP | 0,99 | 3,35 | NA | 0,58 | 1,61 | 12 | 0,99 | 3,55 | 4 | 0,62 | 1,68 | 11 | 0,98 | 3,6 | 5 |
| SP | 0,98 | 3,38 | NA | 0,79 | 2,05 | 8 | 0,97 | 2,98 | NA | 0,96 | 2,81 | NA | 0,99 | 3,99 | NA |

Supplementary table S2 – Statistical power analysis performed using G power software for comparisons of tension variation within groups.

Considered acceptable statistical power if power > 0,75; Alpha error probability = 0,05
