# Supplementary material for: Mechanical consequences at the tendon-bone interface of different medial row knotless configurations and lateral row tension in a simulated rotator cuff repair
Source: J Exp Orthop. 2022 Sep 19;9:94. doi: 10.1186/s40634-022-00536-1 (PMC9482894; doi:10.1186/s40634-022-00536-1)
Supplement: Supplementary file 3 — Additional file 3: Supplementary Table S3. Mean Comparison between locked medial anchor (DP) and sliding medial anchors (SLDP) regarding contact force, area and pressure, peak force and MBR force in the repair box (25N lateral tension) - * reached statistical significance. Supplementary Table S4. Mean comparison between locked medial anchor (DP) and sliding medial anchors (SLDP) regarding contact force, area and pressure, peak force and MBR force in the repair box (50N lateral tension) - * reached statistical significance. Supplementary Table S5. Mean comparison between tape double-hole passage (DP) and single-hole passage (SP) regarding contact force, area and pressure, peak force and MBR force in the repair box (25N lateral tension) - * reached statistical significance. Supplementary Table S6. Mean comparison between tape double–hole passage (DP) and single-hole passage (SP) regarding contact force, area and pressure, peak force and MBR force in the repair box (50N lateral tension) - * reached statistical significance. Supplementary Table S7. Variation within each group if lateral row tension increases 100% - * reached statistical significance. [file 40634_2022_536_MOESM3_ESM.docx]

TABLES

| **Lateral Tension 25N** | DP | Range (+/-) | SLDP | Range (+/-) | Mann Whithney (p<0.05) |
| --- | --- | --- | --- | --- | --- |
| Box Force (N) | **29.68** | 2.77 | **19.44** | 6.57 | **0.032*** |
| Box Area (mm^2^) | 365.00 | 26.72 | 342.40 | 55.15 | 0.548 |
| Box pressure (Mpa) | **0.0815** | 0.01 | **0.0558** | 0.01 | **0.008*** |
| Box peak force (N) | 2.7214 | 0.62 | 1.6801 | 0.70 | 0.056 |
| Box MBR force (N) | 3.417 | 0.89 | 2.22 | 0.77 | 0.095 |

Supplementary Table S3 – Mean Comparison between locked medial anchor (DP) and sliding medial anchors (SLDP) regarding contact force, area and pressure, peak force and MBR force in the repair box (25N lateral tension) - * reached statistical significance

| **Lateral Tension 50N** | DP | Range (+/-) | SLDP | Range (+/-) | Mann Whithney (p<0.05) |
| --- | --- | --- | --- | --- | --- |
| Box Force (N) | 42.52 | 4.05 | 37.44 | 3.80 | 0.151 |
| Box Area (mm^2^) | 420.20 | 11.82 | 412.80 | 27.97 | 0.69 |
| Box pressure (Mpa) | 0.1012 | 0.01 | 0.0929 | 0.01 | 0.151 |
| Box peak Force (N) | 2.7788 | 0.25 | 2.7088 | 0.50 | 1 |
| Box MBR force (N) | 4.67 | 0.75 | 4.46 | 0.43 | 0.421 |

Supplementary Table S4 – Mean comparison between locked medial anchor (DP) and sliding medial anchors (SLDP) regarding contact force, area and pressure, peak force and MBR force in the repair box (50N lateral tension) - * reached statistical significance.

| **Lateral Tension 25N** | DP | Range (+/-) | SP | Range (+/-) | Mann Whithney (p<0.05) |
| --- | --- | --- | --- | --- | --- |
| Box Force (N) | 29.68 | 2.77 | 24.60 | 7.49 | 0.222 |
| Box Area (mm^2^) | 365.00 | 26.72 | 354.5 | 31.34 | 0.421 |
| Box pressure (Mpa) | 0.0815 | 0.01 | 0.0698 | 0.02 | 0.31 |
| Box peak Force (N) | 2.7214 | 0.62 | 2.2951 | 0.40 | 0.31 |
| Box MBR force (N) | 3.42 | 0.89 | 2.66 | 0.66 | 0.222 |

Supplementary Table S5 – Mean comparison between tape double-hole passage (DP) and single-hole passage (SP) regarding contact force, area and pressure, peak force and MBR force in the repair box (25N lateral tension) - * reached statistical significance

| **Lateral Tension 50N** | DP | Range (+/-) | SP | Range (+/-) | Mann Whithney (p<0.05) |
| --- | --- | --- | --- | --- | --- |
| Box Force (N) | 42.52 | 4.05 | 45.90 | 6.25 | 0.31 |
| Box Area (mm^2^) | 420.20 | 11.82 | 416.00 | 28.53 | 0.69 |
| Box pressure (Mpa) | 0.1012 | 0.01 | 0.1103 | 0.01 | 0.222 |
| Box peak Force (N) | **2.7788** | 0.25 | **3.1791** | 0.19 | **0.032*** |
| Box MBR force (N) | 4.67 | 0.75 | 5.14 | 0.58 | 0.421 |

Supplementary Table S6 – Mean comparison between tape double–hole passage (DP) and single-hole passage (SP) regarding contact force, area and pressure, peak force and MBR force in the repair box (50N lateral tension) - * reached statistical significance

| Variation % | DP | Wilcoxson (p<0.05) | SLDP | Wilcoxson (p<0.05) | SP | Wilcoxson (p<0.05) |
| --- | --- | --- | --- | --- | --- | --- |
| Force (N) | **43%** | **0.008*** | **93%** | **0.008*** | **87%** | **0.008*** |
| Area (mm^2^) | **15%** | **0.008*** | 21% | 0.056 | **27%** | **0.016*** |
| Pressure (Mpa) | **24%** | **0.032*** | **66%** | **0.008*** | 43% | 0.095 |
| Peak force (N) | 2% | 0.841 | 61% | 0.056 | **39%** | **0.008*** |
| MBR force (N) | 37% | 0.056 | **102%** | **0.008*** | **93%** | **0.008*** |

Supplementary Table S7 – Variation within each group if lateral row tension increases 100% - * reached statistical significance
